# Assessing Continued Employability among People with Mental Illnesses: Development of a Scale in Japan

**DOI:** 10.3390/ijerph192214786

**Published:** 2022-11-10

**Authors:** Yoshitomo Fukuura, Yukako Shigematsu, Yumi Mizuochi, Tatsuyuki Kakuma

**Affiliations:** 1 Department of Nursing, Kurume University Graduate School of Medicine, 777-1 Higashikushiharamachi, Kurume-shi 830-0003, Fukuoka, Japan; 2 School of Nursing, Kurume University, 777-1 Higashikushiharamachi, Kurume-shi 830-0003, Fukuoka, Japan; 3 The Biostatistics Center, Kurume University, 777-1 Higashikushiharamachi, Kurume-shi 830-0003, Fukuoka, Japan

**Keywords:** mental illness, continued employability, assessment, scale development

## Abstract

Continued employment enables people with mental illnesses to maintain and improve their mental health, and its mutual understanding between them and their workplaces can help provide specific support and improve the work environment. Hence, this study developed a Continued Employability Scale to provide people with mental illnesses solutions for achieving continued employment and examined the scale’s reliability and validity. It is based on a conceptual analysis of the skills necessary for continued employment and comprises items related to continued employability and the consequences of continued employment. We performed conceptual analyses to prepare the item list, conducted the study using a questionnaire survey, and examined its content validity and reliability using factor analyses. The results showed that the developed scale, which can determine self-management, dedication to work, environmental adjustments, and expression of sincerity necessary for continued employment, was reliable and valid. This can be a potentially helpful tool for assessing the ability to continue working and help people with mental illnesses visualise their continued employability, clarify what is being assessed, and improve the self-management ability necessary for continued employment. Further, it can help people who support them at work and enable existing support and programs to function effectively.

## 1. Introduction

Individual placement and support programs designed to help people with mental illnesses remain employed have recently been made available in developed countries and enabled people with mental illnesses to obtain secure employment [1,2,3]. While continued employment is considered vital for enabling people with mental illnesses to maintain and improve their mental health [4,5,6], how to enable them to continue working is still unclear [7,8,9].

Continued employment is more effective for people with mental illnesses than any other medical or social intervention for continuing a healthy lifestyle [10,11,12]. Additionally, support for continued employment improves individual health status and also helps improve the work environment [13], making such support an essential endeavour for both the individual and the workplace. Although continued and secure employment remains challenging for people with mental illnesses, no study has focused on this issue.

Hence, considering the need to examine the continued employability of people with mental illnesses, this study performed a conceptual analysis regarding the skills necessary for continued employment. The analysis revealed that the work ability of people with mental illnesses develops through a reiterative process comprising the following four abilities associated with continued employment: the ability to self-manage, adaptability, the ability to dedicate oneself to work, and the ability to formulate plans. This study also demonstrated that individual and workplace antecedents are the foundation for these abilities. The consequences of continued employment include attaining independence, developing interpersonal relationships, improving and maintaining self-care, achieving happiness, and positive self-transformation [14].

In addition, the above-described structure is only a working hypothesis of the ability to continue working; therefore, the continued employability of people with mental illnesses must be verified with an empirical study conducted with such people who are actually working. Determining specific indicators for measuring continued employability can enable people with mental illnesses and those supporting them to recognise the skills necessary for continued employment, take the necessary steps toward this goal, and contribute to a suitable work environment. Failing to visualise the skills that people with mental illnesses need for continued employment could obscure attributes that need to be addressed, such as who can provide adequate support in which manner and what should be improved, thereby hindering efforts to help people with mental illnesses work continuously. In other words, working toward the goal of enabling continued employment for people with mental illnesses requires visualisation of continued employability to enable themselves to examine solutions for achieving continued employment.

Developing a scale to assess the continued employability of people with mental illnesses can help improve individual efforts regarding work and the work environment. Therefore, this is the first study to develop a measure of continued employability to enable people with mental illnesses to work continuously and examined the reliability and validity of the measure.

## 2. Materials and Methods

The Continued Employability Scale was developed following these steps: (1) Preparation of an item pool, (2) Examination of content validity and revision of items, (3) Application of the scale, (4) Exploratory factor analysis, (5) Examination of scale reliability, and (6) Examination of construct validity and criterion validity.

### 2.1. Definitions of Terms

The continued employability of people with mental illnesses was defined as “their ability to work and dedicate themselves to the job, in cognizance of their mental symptoms and the surrounding environment, while performing planned actions based on proactive self-assessment” [14]. This definition was used as the measurement concept for the Continued Employability Scale.

### 2.2. Continued Employability Scale Draft Development Process

#### 2.2.1. Preparation of an Item Pool

First, we performed a conceptual analysis based on 31 articles on work among people with mental illnesses [14], yielding 338 descriptions. After organising this data, we defined 13 items related to continued employability and 16 items related to the consequences of continued employment. Subsequently, following the literature synthesis, brainstorming, and multiple examinations and revisions for semantic content duplication and accurate expressions in all items, we produced a draft of the Continued Employability Scale, which comprised 35 items related to continued employability and 38 items related to consequences of continued employment.

#### 2.2.2. Examination of Content Validity and Revision of Scale Draft

We quantitatively examined content validity by conducting a questionnaire survey regarding the suitability of the scale draft with five researchers familiar with support for the employment of people with mental illnesses and five people engaged in providing such support.

The suitability of items was assessed with the Content Validity Index [15], wherein a score of ≥0.80 signifies suitability. After eliminating items not confirmed to be suitable and revisions based on experts’ advice, 21 items related to continued employability and 27 items related to the consequences of continued employment remained (see Table 1). These items comprised the revised draft of the Continued Employability Scale. We also asked these 10 individuals for their advice as experts. For all 48 items in the questionnaire, a higher score is considered better and indicates greater continued employability. As such, reverse scoring was not necessary.

### 2.3. Participants

We randomly selected 400 disability support centres for people with mental illnesses across different regions in Japan and included those aged 20 to 59 years registered with the given centres and working as of 1 April 2022.

Each centre was mailed questionnaires for five people, and centre supervisors were asked to pass these questionnaires to people with mental illnesses who were working. Questionnaires were collected either through mail or online using Google Forms.

It is difficult to determine a suitable sample size when factor analysis is conducted. In addition, DeVellis [16] states that for factor analysis including ≤40 items, a sample size of 200 is suitable. Therefore, since the items included in the factor analysis comprised 21 items related to continued employability in the revised draft of the Continued Employability Scale, we sought to obtain data for at least 200 people.

### 2.4. Questionnaire Structure

The basic characteristics in the questionnaire survey consisted of age, sex, diagnosis, clinic visits (yes/no), use of psychiatric medication (yes/no), the number of leaves of absence at current job, and career change (yes/no).

Responses were provided on the following five-point Likert scale: “True (5 points)”, “Somewhat true (4 points)”, “Neither true nor untrue (3 points)”, “Somewhat untrue (2 points)”, and “Untrue (1 point)”.

### 2.5. Analysis Methods

Statistics were analysed with SPSS ver. 28.0 for Windows, as described below.

#### 2.5.1. Item and Factor Analyses

After confirming normality for each item on the Continued Employability Scale and ceiling and floor effects, we determined the number of factors in the initial factor analysis based on changes in initial eigenvalues, a scree plot, and the cumulative contribution ratio. Subsequently, we performed exploratory factor analysis with the stepwise method and Promax rotation. Later, we confirmed the item-total correlation [15] and conducted factor analysis again.

#### 2.5.2. Examination of the Reliability

We examined reliability by calculating Cronbach’s α, which represents internal consistency, for the overall scale and each factor. A Cronbach’s α ≥ 0.7 is considered tolerable, while a Cronbach’s α of ≥0.95 signifies the presence of highly redundant items and is regarded with caution [16].

#### 2.5.3. Examination of Validity

Validity was examined for construct validity and criterion validity. Construct validity was examined by analysing factor structure based on the conceptual analysis content [14] and the characteristics of people with mental illnesses. Regarding criterion validity, career change (yes/no) and consequences of continued employment were considered external criteria potentially related to continued employability; career change was measured with discriminant validity, while consequences of continued employment were measured with predictive validity. First, we used the *t*-test to examine the association of career change as an external criterion with continued employability. Subsequently, due to treating consequences of continued employment as external criteria, we conducted a factor analysis of items related to consequences of continued employment in the main study with the stepwise method. This factor analysis abstracted the following subscales: attainment of social independence, fulfilment in social roles, happiness in daily living, maintenance or improvement of self-care, and growth in interactions. Studies have shown that sustainable employment yields health, happiness, and productivity and improves relationships with one’s family, co-workers, superiors, and employers [1,17]. The factor analysis results showed the items relating to consequences of continued employment, composed of the categories of attaining independence in work, achieving happiness, positive self-transformation, establishing human connections, and maintaining or improving self-care, demonstrated in a conceptual analysis in a previous study [14]. Therefore, the consequences of continued employment external criteria were considered to examine the criterion validity of the Continued Employability Scale and determine the Pearson product-moment correlation coefficient between the scores for consequences of continued employment score and employability.

### 2.6. Ethical Considerations

This study was conducted with the approval of the Kurume university (approval number 21306).

Representatives from the participants’ centres were provided with a written explanation of the study’s outline and methods, ethical considerations for the participants, and the publication of study results. Centre representatives distributed participation request forms and survey forms to participants. The survey forms were anonymised to prevent the identification of individual participants. The participation request form explained to participants the voluntary nature of participation, that not participating would not confer any disadvantage in their jobs, and that responding to the survey either by filling in the survey form or completing the online survey would constitute consent to participate in the study. It was also explained that due to pseudonymisation (to prevent identification of specific individuals), surveys could not be destroyed following collection, and data could not be deleted.

## 3. Results

### 3.1. Participants’ Overview

The total number of participants was 296. The participants comprised 155 men (53.08%) and 137 women (46.92%). Regarding age, 39 participants (13.54%) were in their twenties, 68 (23.61%) were in their thirties, 99 (34.38%) were in their forties, and 82 (28.47%) were in their fifties. Diagnoses consisted of schizophrenia in 110 participants (37.67%), depression in 61 participants (20.89%), bipolar disorder in 29 participants (9.93%), obsessive compulsive disorder in 6 participants (2.05%), autism in 42 participants (14.38%), alcoholism in 3 participants (1.03%), and other in 41 participants (14.04%). Regarding clinic visits, 285 participants (96.61%) responded “yes”, while 10 (3.39%) responded “no”. Psychiatric medication was used by 257 participants (87.07%) and not used by 38 participants (12.93%). Regarding leave of absence from their current job, 206 participants (69.59%) responded “no”, while 90 participants (33.46%) responded “yes”. When asked if they had ever changed careers, 69 participants (23.47%) responded with “no”, while 225 participants (76.53%) responded “yes” (Table 2). The total number of characteristics excluded those who did not respond to that characteristic.

### 3.2. Examination of the Continued Employability Scale

Examination of the score distributions for the 21 items of the Continued Employability Scale revealed that score distributions were uneven for several items. In addition, a close inspection of the items’ content with uneven score distributions revealed that all items in question were indispensable for measuring the concept of continued employability. Therefore, we chose not to exclude any of these items and included them in subsequent analyses.

Subsequently, we performed factor analysis for the 21 scale items with the stepwise method. Eigenvalues changed in the following manner: 7.46, 2.21, 1.32, 1.10, while the slope of the factor scree plot suggested that a four-factor structure was valid. Therefore, assuming four factors, we performed factor analysis again with the stepwise method and Promax rotation. Two items that did not demonstrate sufficient factor loading were excluded from the analysis, and we performed factor analysis again with the stepwise method and Promax rotation. Table 3 and Table 4 show the final factor pattern following Promax rotation and correlations between factors. The four post-rotation factors explained 49.87% of the total variance in the 19 items.

The examination of the reliability of the Continued Employability Scale revealed that the overall Cronbach’s α was 0.894. Cronbach’s α values for Factor 1 (self-management), Factor 2 (dedication to work), Factor 3 (adjustments in the environment), and Factor 4 (expression of sincerity) were 0.792, 0.793, 0.767, and 0.785, respectively; all of these were ≥0.7, confirming that internal consistency had been achieved (Table 3).

Factor 1 comprised ten items. Items that involved facing work with an objective appraisal of one’s symptoms and condition and adjusting oneself accordingly (e.g., “I turn down requests from others because of my symptoms”, “I tailor the way I work to my symptoms”, “When I am tired from work, I re-examine my lifestyle (diet, sleep, etc.) to recover from fatigue”, etc.) demonstrated high loading. Therefore, we named Factor 1 “self-management”.

Factor 2 comprised four items. Items that involved completing assigned work (e.g., “I focus on the job at hand”, “I take on work enthusiastically”, “I stick with a set task until the end”, etc.) demonstrated high loading. Therefore, we named Factor 2 “dedication to work”.

Factor 3 comprised three items. Items that involved methods for enabling oneself to continue working (e.g., “A supervisor or leader adjusts my working hours”, “My work is tailored to my symptoms”, “I show my strong points at work”, etc.) demonstrated high loading. Therefore, we named Factor 3 “adjustments in environment”.

Factor 4 comprised two items. Items that involved behaviours as a member of society in interpersonal relationships (e.g., “I apologise for my mistakes”, “I convey my gratitude when someone helps me with work”, etc.) demonstrated high loading. Therefore, we named Factor 4 “expression of sincerity”.

Table 4 shows the correlations between the subscales of the Continued Employability Scale. The four subscales demonstrated significant positive correlations (Table 4).

### 3.3. Criterion Validity

To examine criterion validity, we calculated the correlation coefficients between (1) career change (yes/no) and the Continued Employability Scale and (2) consequences of continued employment and the Continued Employability Scale.

#### 3.3.1. Association between a Career Change and Continued Employability Scale

Table 5 shows the association between a career change and the Continued Employability Scale (Table 5).

#### 3.3.2. Association between Consequences of Continued Employment and Continued Employability Scale Score

The association between the consequences of continued employment and the Continued Employability Scale score is shown in Table 6.

Table 7 shows the correlations between Continued Employability Scale subscales and subscales for consequences of continued employment. All four Continued Employability Scale subscales were significantly correlated with all subscales of consequences of continued employment (Table 7).

## 4. Discussion

This study aimed to develop a scale to measure continued employability among people with mental illnesses and examine how to support them as they strive for continued employment. We examined the reliability and validity of the Continued Employability Scale, support for continued employability, and the application of the scale.

### 4.1. Reliability and Validity of the Continued Employability Scale

Cronbach’s α for the 19 items of the Continued Employability Scale was 0.894, while Cronbach’s α for each subscale was ≥0.75, signifying sufficient reliability.

Subsequently, we examined the construct validity of the Continued Employability Scale for people with mental illnesses. The scale was confirmed to consist of a four-factor structure: self-management, dedication to work, adjustments in the environment, and expression of sincerity. Factor 1, self-management, showed the importance for people with mental illnesses to objectively view their own symptoms and condition and make necessary adjustments, making this the most crucial of the four factors. Self-management was highly correlated with all five subscales of consequences of continued employment (attaining independence, developing interpersonal relationships, improvement and maintenance of self-care, achieving happiness, and positive self-transformation), demonstrating the importance of self-management. Furthermore, self-management corresponds to the ability to self-manage, as found in an earlier conceptual analysis [14], to be involved with the continuation of work, and was the core of continued employability. Additionally, self-management improvement betters both job performance and self-efficacy [18,19], suggesting that self-management affects various elements of work continuation.

Factor 2, dedication to work, shows the importance of focus in one’s work. People with mental illnesses characteristically have difficulty focusing on their work due to their mental symptoms [20]. Workers must perform their duties while dealing with risks as they strive for their workplace benefits and their own independence [14,21], an important aspect of which is to focus on work with enthusiasm. Regardless of mental impairments, dedication to work may be an indispensable ability for continued employment.

Factor 3, adjustments in the environment, shows the importance of people with mental illnesses having others at their workplace adjust their duties according to their symptoms. This factor corresponds to the ability to adapt, abstracted in our previous conceptual analysis [14], and formed the foundation of continued employment alongside self-management. Workers who suffer severe psychosocial stress at work may quit before experiencing a deterioration in their health condition and are forced to take sick leave; notably, working arduously may exacerbate this stress [9]. People with mental illnesses require the cooperation of those around them to avoid the difficulties posed by their symptoms as they perform their duties [22]. Therefore, when changes in symptoms hinder work, people with mental illnesses may need to consult with others at their workplace and adjust their environment to continue working.

Factor 4, expression of sincerity, wherein gratitude and apology were abstracted, was positively correlated with the five items of consequences of continued employment and vital for continued employment. Expression of sincerity builds smooth interpersonal relationships through gratitude and apology and is a crucial social skill for solving problems at work [7,23]. To continue working, people with mental illnesses must cooperate with their workplaces’ human resource departments and mental health teams [24] and manage their own workspace to enable them to control their work [25]. Therefore, understanding the workplace environment and personal coping strategies are necessary for continued employment. Thus, while self-management and environmental adjustments are crucial for continued employment, building interpersonal relationships that enable such adjustments may also be important. This study demonstrated that in addition to self-management and adjustments in the environment, expression of sincerity is also crucial for continued employment; specifically, work was shown to require gratitude, apology, and other basic social skills to enable smooth self-management and adjustments in the environment.

Therefore, for people with mental illnesses to work continuously, they must adjust their environment while expressing sincerity with a foundation of self-management and dedicate themselves to their work with the cooperation of those around them to avoid taking on more than they can handle. Additionally, the ability to formulate plans, as found in our previous conceptual analysis [14], was included in Factor 1 (self-management) and was not abstracted as a factor in the present study. Planning may have been treated as an ability exercised under self-management. In summary, the factor structure, which comprised self-management, dedication to work, adjustments in the environment, and expression of sincerity, was determined to be valid. Moreover, the Continued Employability Scale developed in this study demonstrated positive correlations with the above and among its subscale factors, indicating that construct validity was achieved.

Subsequently, we examined the criterion validity of the four-factor, 19-item Continued Employability Scale with career change (yes/no) and consequences of continued employment as external criteria. The Continued Employability Scale score differed significantly based on whether participants had changed careers and was positively correlated with consequences of continued employment, supporting the criterion validity of the scale.

Based on the above, the Continued Employability Scale, developed to measure the potential for people with mental illnesses to work continuously without leaves of absence, was determined to be reliable and valid, signifying that its factor structure is valid as a scale for assessing continued employability.

### 4.2. Application of the Continued Employability Scale

The scale developed in this study for assessing continued employability among people with mental illnesses was confirmed to be reliable and valid. The scale, which can determine self-management, dedication to work, adjustments in the environment, and expression of sincerity necessary for continued employment, is a valuable and applicable tool for assessing the ability to continue working. People with mental illnesses can use the scale to reflect on what they need to work on continuously. Such reflection could enable them to realise the abilities they need and think about what they should do to improve their ability to work. Objective and subjective assessment can improve job performance [18]. Therefore, allowing people with mental illnesses to visualise their own continued employability clarifies what is being assessed and can help improve the self-management ability necessary for continued employment.

In addition, people with mental illnesses cannot attain this ability with assessment alone. People with mental illnesses are limited in how much they can manage themselves and, therefore, require understanding and support, such as through consultation with others, to continue working. As demonstrated in our previous conceptual analysis [14], a workplace that believes in the employees’ abilities, has a support system, and a sense of security is an essential prerequisite for continued employment [26,27,28]. For example, Factor 3, adjustments in the environment, was characterised by people with mental illnesses appealing to supervisors and colleagues to adjust their environment to continue working. As such adjustments may not be achieved merely by people with mental illnesses asking for them at their workplaces personally, Factor 3 is predicated on the idea that the work environment already enables adjustments and the understanding of others. A work environment without sufficient understanding of the work ability of people with mental illnesses is predicted to increase their burden. Therefore, a shared understanding of continued employability between people with mental illnesses and their workplaces may provide an opportunity to re-examine specific, necessary support and the work environment and could help improve the work environment. Care from staff both within and outside businesses already exists as employee assistance programs, comprising both care for individual productivity and systematic care [29,30,31]. In the United Kingdom, employment advisors provide decision-making support for job adjustment, and initiatives have been undertaken to achieve desired outcomes [32]. Additionally, active listening, which involves paying attention to the speaker, understanding them, and sympathising with them, is crucial in interpersonal relationships for many jobs [33]. Thus, a deeply rooted culture wherein people with mental illnesses can seek help, and the workplace provides organised support may be critical for continued employment.

The above indicates that the Continued Employability Scale allows both people with mental illnesses and those who support them at work to visualise the ability to continue working and can therefore be applied as an accessory tool to enable existing support and programs to function effectively.

## 5. Conclusions

This study developed a Continued Employability Scale to enable people with mental illnesses to continue working without taking a leave of absence from work for their mental health. Analysis of the reliability and validity of the scale demonstrated the following.

1. The scale, which comprised 19 items across four factors (self-management, dedication to work, adjustments in the environment, and expression of sincerity), was confirmed to be reliable and valid.
2. The four subscale factors of continued employability and the five subscale factors of consequences of continued employment were all correlated with each other, while continued employability was associated with career change (yes/no).
3. People with mental illnesses can apply the Continued Employability Scale to understand how to generally perform in their jobs and allow their co-workers to understand the characteristics of people with mental illnesses and support interactions with them. The scale can also be applied as an accessory tool to bolster existing support.

### Limitations and Future Prospects

This study’s results were affected by employment support for people with mental illnesses and the employment environment in Japan. Therefore, our results may not apply to other countries with different systems. Additionally, our results came from responses to surveys sent to people with mental illnesses registered with disability support centres throughout Japan, indicating that the participants may have been relatively enthusiastic about working. People with mental illnesses who work continuously may not have been randomly sampled from the general population. Additionally, although continued employability is affected by the work environment, our results did not account for the effects of the work environment; therefore, the association between the two must be analysed and investigated.

To obtain results that reflect the general population of people with mental illnesses who work continuously without taking a leave of absence, future studies can continue collecting data to refine our scale while analysing the relation between the prerequisites yielded in conceptual analysis and continued employability in our Continued Employability Scale.

## Figures and Tables

**Table 1:** Questionnaire on continued employability and the consequences of continued employment.

Continued Employability
(1) I confirm the schedule for the day
(2) I focus on the task at hand
(3) I take on work enthusiastically
(4) I ask what I do not understand
(5) I stick with a set task until the end
(6) I adjust my environment to make it conducive to working
(7) I think positively even when I make a mistake at work
(8) I consult with my co-workers about work-related troubles and other matters
(9) I convey my gratitude when someone helps me with work
(10) I apologise for my mistakes
(11) A supervisor or leader adjusts my working hours
(12) I show my strengths at work
(13) My work is tailored to accommodate my symptoms
(14) I am aware of stress from work and think about what I can do about it
(15) When I am tired from work, I re-examine my lifestyle (diet, sleep, etc.) to recover from fatigue
(16) I turn down requests from others because of my symptoms
(17) I avoid personal stressors (rumours, whispers, etc.)
(18) I engage in activities (exercise, video games, etc.) to relieve stress from work
(19) I tailor the way I work to accommodate my symptoms
(20) I think about the factors that exacerbate my symptoms
(21) I check the progress of work around me
**Consequences of continued employment**
(1) I make decisions for myself
(2) I use my free time meaningfully
(3) I live independently from my family
(4) I am useful to society
(5) I follow through completely in doing my part
(6) I have more social interactions
(7) I have more occasions to talk with family and friends
(8) I am coping with my symptoms
(9) I wake up when I need to
(10) I get a good night’s sleep
(11) I manage my time well
(12) I feel fulfilled at work
(13) I feel value in working
(14) I have gained confidence in myself
(15) I now have a goal
(16) I am satisfied with my life
(17) I have “ikigai” (a sense of purpose, a reason for living)
(18) I do not feel isolated
(19) I am accepted by others
(20) I am valued
(21) I take pride in the fact that I am working
(22) I feel I have grown as a person
(23) I reflect on my past
(24) I have learned new things
(25) I am ambitious
(26) I think positively about the future
(27) I feel satisfied

**Table 2:** Basic characteristics.

Characteristic	Category	N	%
Sex (n = 292)	Male	155	53.08
	Female	137	46.92
Age (n = 288)	20–29	39	13.54
	30–39	68	23.61
	40–49	99	34.38
	50–59	82	28.47
Diagnosis (n = 292)	Schizophrenia	110	37.67
	Depression	61	20.89
	Bipolar disorder	29	9.93
	Obsessive compulsive disorder	6	2.05
	Autism	42	14.38
	Alcoholism	3	1.03
	Other	41	14.04
Clinic visits (n = 295)	Yes	285	96.61
	No	10	3.39
Use of psychiatric medication (n = 294)	Yes	256	87.07
	No	38	12.93
Leaves of absence at current job (n = 296)	Yes	90	33.46
	No	206	69.59
Career change (n = 294)	Yes	225	76.53
	No	69	23.47

**Table 3:** Continued Employability Scale factor analysis results (factor pattern following promax rotation) *.

	I	II	III	IV	Factor Loading
Factor 1: Self-management (Cronbach’s α = 0.792)					
(19) I tailor the way I work to accommodate my symptoms	0.80	−0.08	0.12	−0.08	0.65
(16) I turn down requests from others because of my symptoms	0.75	−0.15	−0.03	−0.01	0.44
(15) When I am tired from work, I re-examine my lifestyle (diet, sleep, etc.) to recover from fatigue	0.68	0.09	−0.11	0.03	0.47
(20) I think about the factors that exacerbate my symptoms	0.66	0.00	−0.14	0.10	0.40
(18) I engage in activities (exercise, video games, etc.) to relieve stress from work	0.57	−0.05	0.06	−0.07	0.30
(14) I am aware of stress from work and think about what I can do about it	0.50	0.05	0.17	0.05	0.44
(8) I consult with my co-workers about work-related troubles and other matters	0.49	−0.05	0.16	0.15	0.41
(17) I avoid personal stressors (rumours, whispers, etc.)	0.48	−0.05	−0.12	0.16	0.23
(7) I think positive even when I make a mistake at work	0.46	0.27	0.01	−0.06	0.40
(6) I adjust my environment to make it conducive to working	0.41	0.39	0.01	−0.08	0.46
Factor 2: Dedication to work (Cronbach’s α = 0.793)					
(2) I focus on the task at hand	−0.13	0.91	0.00	−0.02	0.68
(3) I take on work enthusiastically	0.07	0.78	−0.08	0.00	0.64
(5) I stick with a set task until the end	−0.12	0.72	0.01	0.16	0.58
(1) I confirm the schedule for the day	0.02	0.47	0.08	0.04	0.28
Factor 3: Adjustment of environment (Cronbach’s α = 0.767)					
(11) A supervisor or leader adjusts my working hours	−0.18	−0.07	0.92	0.09	0.70
(13) My work is tailored to accommodate my symptoms	0.19	−0.03	0.66	0.00	0.60
(12) I show my strengths at work	0.18	0.22	0.48	−0.13	0.47
Factor 4: Expression of sincerity (Cronbach’s α = 0.785)					
(10) I apologise for my mistakes	−0.03	0.05	0.09	0.79	0.70
(9) I convey my gratitude when someone helps me with work	0.15	0.11	−0.04	0.66	0.63

* Overall Cronbach’s α for Continued Employability Scale = 0.894.

**Table 4:** Correlations among factors in continued employability.

	I	II	III	IV
Factor 1: Self-management	1	-	-	-
Factor 2: Dedication to work	0.488 **	1	-	-
Factor 3: Adjustments in environment	0.547 **	0.285 **	1	-
Factor 4: Expression of sincerity	0.460 **	0.539 **	0.281 **	1

Pearson correlation; ** *p* < 0.01.

**Table 5:** Association between career change and Continued Employability Scale.

	Continued Employability	
	Mean	Standard Deviation
Yes	80.16	12.94
No	84.51	12.58
Mann–Whitney U test **		

** *p* < 0.01.

**Table 6:** Correlation between Continued Employability Scale and consequences of continued employment.

	Overall Consequences of Continued Employment Score	Overall Continued Employability Score
Overall consequences of continued employment score	1	
Overall continued employability score	0.779 **	1

Pearson correlation; ** *p* < 0.01.

**Table 7:** Correlations between Continued Employability Scale subscales and consequences of continued employment subscales.

		Continued Employability Scale			
		Self-Management	Dedication to Work	Adjustments in Environment	Expression of Sincerity
	Attaining independence	0.693 **	0.571 **	0.461 **	0.434 **
	Fulfilment in social roles	0.505 **	0.545 **	0.397 **	0.416 **
Consequences of continued employment	Achieving happiness	0.581 **	0.431 **	0.480 **	0.359 **
	Improvement and maintenance of self-care	0.534 **	0.472 **	0.333 **	0.401 **
	Developing interpersonal relationships	0.503 **	0.319 **	0.430 **	0.307 **

Pearson’ correlation; ** *p* < 0.01.

## Data Availability

Not applicable.
